# Breast cancer distant recurrence lead time interval by detection method in an institutional cohort

**DOI:** 10.1186/s12885-020-07609-3

**Published:** 2020-11-20

**Authors:** Henry G. Kaplan, Judith A. Malmgren, Mary K. Atwood

**Affiliations:** 1 Swedish Cancer Institute, 1221 East Madison, Seattle, WA 98104 USA; 2 HealthStat Consulting, Inc., Seattle, WA USA; 3 School of Public Health, University of Washington, Seattle, WA USA

**Keywords:** Lead time, Lead time bias, Survival, Breast cancer, Detection, Cox proportional hazards model, Mammography, Early diagnosis

## Abstract

**Background:**

Lead time, the interval between screen detection and when a disease would have become clinically evident, has been cited to explain longer survival times in mammography detected breast cancer cases (BC).

**Methods:**

An institutional retrospective cohort study of BC outcomes related to detection method (mammography (MamD) vs. patient (PtD)). Cases were first primary invasive stage I-III BC, age 40–74 years (*n* = 6603), 1999–2016. Survival time was divided into 1) distant disease-free interval (DDFI) and 2) distant disease-specific survival (DDSS) as two separate time interval outcomes. We measured statistical association between detection method and diagnostic, treatment and outcome variables using bivariate comparisons, Cox proportional hazards analyses and mean comparisons. Outcomes were distant recurrence (*n* = 422), DDFI and DDSS.

**Results:**

39% of cases were PtD (*n* = 2566) and 61% were MamD (*n* = 4037). MamD cases had a higher percentage of Stage I tumors [MamD 69% stage I vs. PtD 31%, *p* < .001]. Rate of distant recurrence was 11% among PtD BC cases (*n* = 289) vs. 3% of MamD (*n* = 133) (*p* < .001). Order of factor entry into the distant recurrence time interval (DDFI) model was 1) TNM stage (*p* < .001), 2) HR/HER2 status (*p* < .001), 3) histologic grade (*p* = .005) and 4) detection method (*p* < .001). Unadjusted PtD DDFI mean time was 4.34 years and MamD 5.52 years (*p* < .001), however when stratified by stage, the most significant factor relative to distant recurrence, there was no significant difference between PtD and MamD BC. Distant disease specific survival time did not differ by detection method.

**Conclusion:**

We observed breast cancer distant disease-free interval to be primarily associated with stage at diagnosis and tumor characteristics with less contribution of detection method to the full model. Patient and mammography detected breast cancer mean lead time to distant recurrence differed significantly by detection method for all stages but not significantly within stage with no difference in time from distant recurrence to death. Lead time difference related to detection method appears to be present but may be less influential than other factors in distant disease-free and disease specific survival.

## Background

The incidence of recurrent metastatic breast cancer (rMBC) and breast cancer mortality have decreased in recent years coincident with improvement in breast cancer survival due to both reduced incidence of higher stage disease related to mammography screening and improved adjuvant systemic therapy for invasive stage I-III disease [1–4]. Debate and analysis continue about the relative contribution of early detection of breast cancer by mammography screening to improved survival [5–8]. From national mammography screening program surveillance reports, mammography detected tumors are more often smaller and lower stage with better survival [9].

Mammography screening has shifted breast cancer detected to more early stage (stage 0 (DCIS) and stage I-II) and less late stage breast cancer (stage III and IV) over time in screened populations [10–16]. The mechanism behind early detection is the use of mammography imaging to screen asymptomatic women at regular intervals for preclinical disease recognized by screening examination and advancing time of diagnosis by the interval that would otherwise occur for breast cancer detection without screening [17, 18].

Lead time is described as the interval between screening detection and when the disease would have become clinically evident without screening. Lead time gained from screening detection may lengthen the interval when added to the time over which evident disease progresses. Lead time is not a measure used to evaluate breast cancer survival improvement associated with screening mammography which is measured by differential breast cancer mortality over time between screened and unscreened populations.

We are now in a time of accepted validity for mammography screening with evidence-based guidelines adopted and promoted in the United States and Europe [19–21]. Mammography screening is not institutionalized in the United States where it is an opportunistic choice based on health care access, screening guideline knowledge, insurance coverage and care giver recommendation [22].

Timing and incidence of invasive breast cancer distant disease recurrence provides an opportunity to measure lead time by comparing time to distant recurrence after initial diagnosis and post recurrence survival as a function of detection method. In our retrospective institutional cohort study, the objective was to measure time to distant disease recurrence and time from distant recurrence to death among invasive breast cancer patients to assess whether lead time is differential by how the breast cancer was detected.

## Methods

To assess the contribution of lead time to survival among mammography detected BC cases we conducted lead time analysis comparing mammography (MamD) to patient detected (PtD) BC using time to distant recurrence as the first interval (DDFI) and time from distant recurrence to last follow up or death from disease as the second interval (distant disease specific survival (DDSS)) separately and combined. We compared distant recurrence lead time by detection method to DDFI and DDSS, the two component time intervals of disease specific survival, and modeled the relative contribution of detection method to DDFI. We also assessed relative rMBC incidence by detection method.

### Study design

**Fig. 1:**
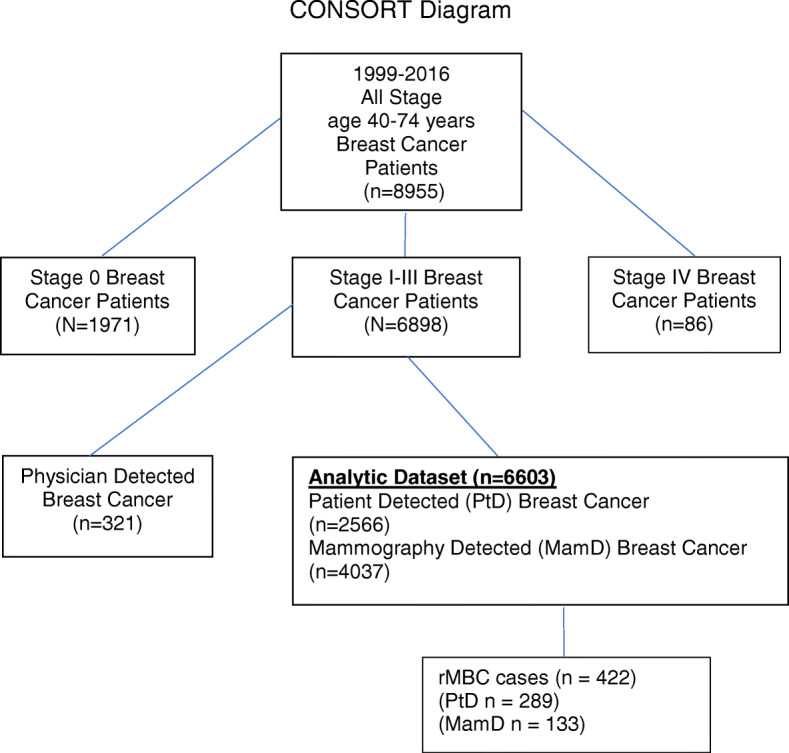
CONSORT diagram

We conducted a retrospective cohort analysis of all first primary stage I-III invasive BC cases age 40–74 from 1990 to 2016, with follow-up through 2018 for distant recurrence and vital status (*n* = 6603). Age 40–74 years was selected based on screening recommendations during this time period [19, 23, 24]. Non-surgical cases (*n* = 18), patients who refused recommended treatment other than surgery (*n* = 24), cases with unknown method of detection (*n* = 11) and cases with unknown cancer status at follow up (*n* = 139) were excluded from the analysis. Inflammatory breast cancer (IBC) (T4) cases were excluded (*n* = 125), as 96% of the IBC cases were patient detected and symptom based not detected by mammography. Patient (PtD) and mammography detected (MamD) BC was included and BC found by a medical professional from a lump or abnormality during routine physical examination was excluded (*n* = 295) (Fig. 1).

Our institutional breast cancer registry database contains detailed information on diagnosis, pathology, staging, treatment, tumor markers, and vital status at follow up including cause-specific death. Incident BC cases are entered at time of diagnosis into the HIPAA compliant and IRB approved registry. Patient vital and disease status including date, site and type of recurrence and date and cause of death are collected prospectively through annual updates by a certified cancer registrar complete through 2018 for this cohort. Follow-up status was obtained from 1) electronic chart review, 2) IRB-approved physician directed follow-up letter, 3) the institution’s cancer registry, and 4) Surveillance Epidemiology and End Results (SEER) Seattle-Puget Sound Registry [25].

Distant disease recurrence (rMBC) was restricted to first presentation of distant disease excluding dates of subsequent disease progression. Hormone receptor positivity was estrogen and/or progesterone receptor positive (HR positive) and HR negative if negative for both. Self-reported race was coded white/non-white. All cases were coded to AJCC 7 classic anatomic staging across all years [26]. TNM stage 0 were excluded from the analysis as few were patient detected and distant recurrence was a rare event. Distant recurrence (rMBC) was designated if distant disease diagnosis occurred 3 months or more post initial diagnosis.

Breast cancer detection method was obtained by medical record review by a certified cancer registrar. Mammography detected was assigned to breast cancer discovered by routine mammography in the absence of complaints or known physical findings or as a repeat or diagnostic mammogram to verify a previous equivocal mammography finding. Patient detection was assigned if the patient presented with personally detected breast symptoms, such as a palpable lump, pain, swelling, nipple discharge, or bleeding which prompted a doctor visit. Patients with self-detected tumors may have subsequently had a mammogram or ultrasound done but would still be categorized as a patient-detected breast cancer from first presentation. Detection method was recorded by the physician at time of diagnosis and was only assigned if it was certain from the record.

Pearson chi-square test comparisons of categorical characteristics by detection method and mean comparisons for continuous variables were used (F statistic). Distant disease-free interval (DDFI) was time from primary BC diagnosis to distant recurrence, distant disease specific survival (DDSS) was time from distant recurrence (rMBC) to last follow-up or death from this disease, and disease specific survival (DSS) was total time from initial BC diagnosis to last follow-up or death from this disease. By dividing DSS into two component parts, time to distant disease recurrence (DDFI) and time from distant disease recurrence to last follow up or death from disease (DDSS), we are able to identify which portion of survival time is affected by lead time and evaluate accordingly. Kaplan-Meier estimation was used to calculate 5-year DDFI, DDSS and DSS rates (log rank tests).

Covariates significant by detection method were used to build the model, informed by the chi-square analysis and tested a priori using stepwise entry. The multivariable Cox proportional hazards model was used to estimate adjusted hazard ratios (HzR) and corresponding 95% confidence intervals (CI) using DDFI as the outcome. We evaluated the proportional hazards assumption by plotting ln{−ln(survival)} curves for the ordinal covariate of diagnosis year versus ln (at risk time) and on the basis of Schoenfeld residuals after fitting individual Cox models. We found no evidence suggesting substantial violation of the proportionality assumption graphically or in tests for interaction with the logarithm of survival time [27]. Effect modification was evident from the Cox proportional hazards analysis with stage the dominant variable in the model. Therefore, lead time analysis was stratified by stage to compare detection method differences in survival [28, 29]. All *p*-values were 2-sided and analyses were conducted using SPSS v.26 [30].

## Results

Between 1999 and 2016, 39% of invasive stage I-III breast cancer cases were patient detected (*n* = 2566) and 61% were mammography detected (*n* = 4037). Sixty nine percent of MamD BC cases were stage I at diagnosis, 27% stage II and 4% stage III. Thirty one percent of PtD BC were stage I at diagnosis, 51% stage II and 18% stage III (*p* < .001). PtD BC patients trended to younger age at diagnosis, 37% age 40–49 years vs. 19% MamD and MamD cases trended older [mean age PtD = 55 years vs MamD = 58 years (*p* < .001)]. More MamD BC cases identified as white race (84% vs 77%) Table 1.

**Table 1:** Descriptive characteristics by detection method 1999–2016 (*n* = 6603)

	PtD	MamD	*p* value
	(*n* = 2566)	(*n* = 4037)	
Stage	N (column %)	N (column %)	
I	792 (31%)	2772 (69%)	<.001
II	1314 (51%)	1108 (27%)	
III	460 (18%)	157 (4%)	
Age			
40–49	950 (55%)	765 (45%)	<.001
50–64	1216 (36%)	2189 (64%)	
65–74	400 (27%)	1083 (73%)	
Mean age (range, F statistic)	54 (40–74)	58 (40–74)	<.001
Race			
White	1986 (37%)	3388 (63%)	<.001
Non-White	580 (47%)	649 (53%)	
Hormone receptor status			
HR+	2081 (36%)	3641 (64%)	<.001
HER2 status			
Her2+ (HR- or HR+)	458 (46%)	534 (54%)	<.001
HR/HER2 status at initial diagnosis			
HR+/HER2-	1718 (36%)	3110 (64%)	<.001
HR+/HER2+	332 (45%)	399 (55%)	
HR−/HER2-	340 (61%)	216 (39%)	
HR−/HER2+	126 (49%)	134 (51%)	
Histologic type initial primary breast tumor			
Ductal	2122 (39%)	3312 (61%)	.287
Lobular	258 (39%)	411 (61%)	
Lobular/Ductal mixed	120 (40%)	177 (60%)	
Other cancer	64 (33%)	133 (67%)	
Nuclear grade initial primary breast tumor			
Low/Intermediate	1189 (31%)	2675 (69%)	<.001
High	1340 (51%)	1289 (49%)	
Histologic grade initial primary breast tumor			
Low/Intermediate	583 (29%)	1447 (71%)	<.001
High	1943 (44%)	2498 (56%)	
Tumor size (mean, range, F statistic)	2.91 (.10, 18.00)	1.51 (.05, 17.00)	<.001
# Positive nodes (mean, range, F statistic)	1.67 (0–44)	.57 (0–35)	<.001
Treatment			
Surgery only	339 (36%)	614 (64%)	<.001
Surgery/radiation	575 (22%)	1997 (78%)	
Surgery/chemotherapy	412 (53%)	372 (47%)	
Surgery/radiation/chemotherapy	1240 (54%)	1054 (46%)	
Distant recurrence			
Yes	289 (68%)	133 (32%)	<.001

Tumor characteristics differed with MamD BC cases more likely HR+/HER2- [64% vs. 36% PtD BC] and the reverse for triple negative breast cancer (TNBC) [39% MamD BC vs 61% PtD BC) (*p* < .001)]. MamD tumors were smaller and more often <= 2 cm in size (77%) vs. 42% of PtD BC cases (*p* < .001) [mean tumor size: MamD = 1.51 cm, PtD = 2.91 cm (*p* < .001)]. Twenty one percent of MamD BC had positive lymph nodes vs 44% of PtD BC patients (*p* < .001). Histologic type was not significantly different. PtD BC nuclear grade was more often high grade than MamD BC (52% vs. 32%). PtD and MamD cases were both majority high grade, PtD 76% and MamD 62% (*p* < .001). 15% or fewer of each group had surgery treatment only, with the majority of MamD BC cases receiving surgery/radiation treatment (50%) and the majority of PtD BC cases receiving surgery/radiation/chemotherapy treatment (54%) (*p* < .001) Table 1.

**Fig. 2:**
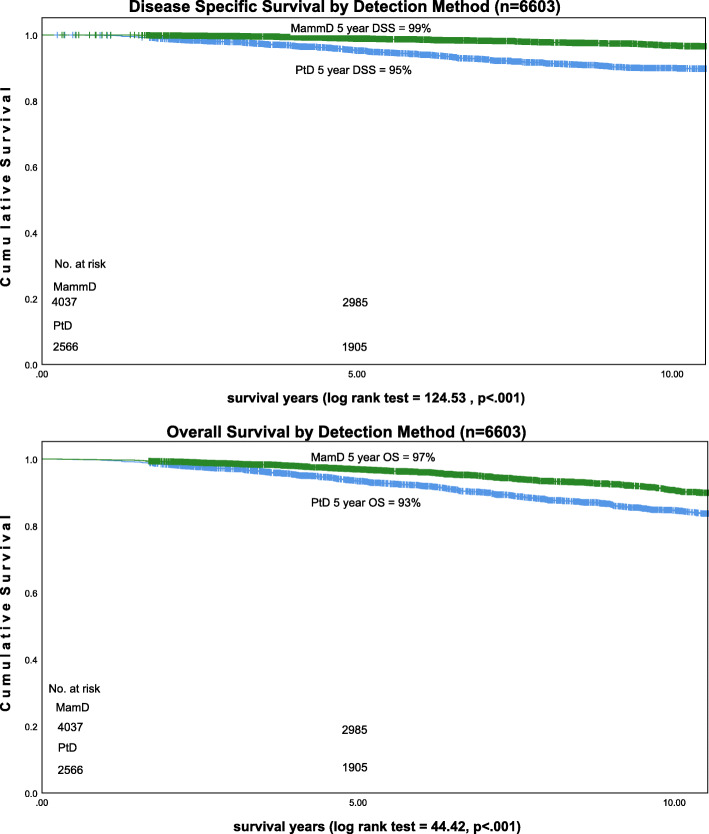
DSS and OS by detection method

Average follow up was 9 years [range 1.7 to 20.4 years]. Eleven percent of PtD BC cases had a distant recurrence (rMBC) (*n* = 289) compared to 3% of MamD BC (*n* = 133). Five-year disease specific survival was 99% for MamD BC and 95% for PtD BC (*p* < .001). Five-year overall survival was 97% for MamD BC and 93% for PtD BC (*p* < .001) Fig. 2.

**Fig. 3:**
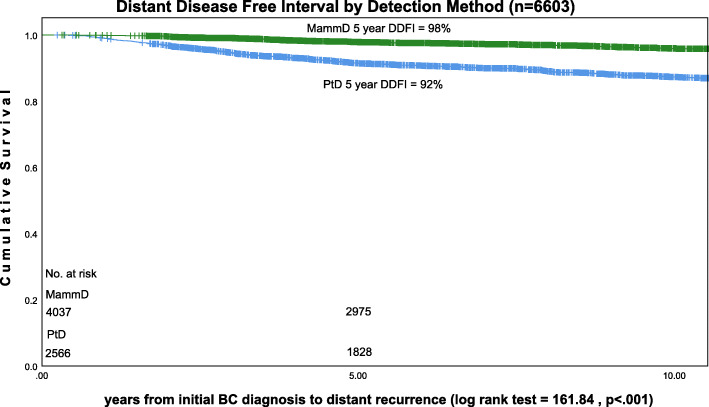
DDFI survival: all cases (*n* = 6603)

**Fig. 4:**
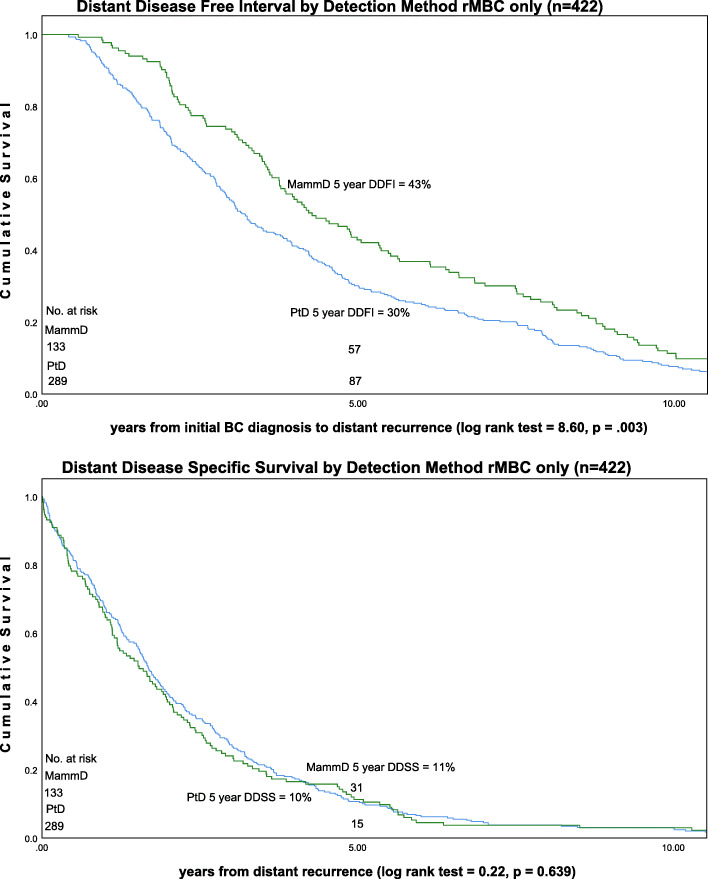
DDFI survival and DDSS by Detection Method: rMBC only (*n* = 422)

Using all cases in the analytic set (*n* = 6333) the distant disease-free interval five-year survival rate was 98% for MamD BC and 92% for PtD BC (*p* < .001) Fig. 3. For the subset of patients with distant recurrence (rMBC = 422), distant disease-free interval five-year survival was 43% for MamD BC and 30% for PtD BC (*p* < .001) [DDFI: time from initial diagnosis to last follow-up or distant disease]. Distant disease specific five-year survival was 11% for rMBC MamD BC and 10% for rMBC PtD BC (not significant) (*n* = 422) [DDSS: time from distant recurrence to death from disease or last follow-up] Fig. 4.

In the forward conditional Cox proportional hazards model of time to distant recurrence (DDFI) using rMBC as the outcome (*n* = 6603), variable order of entry into the model at <.05 significance level was 1) TNM stage 2) HR/HER2 status, 3) histologic grade, and 4) detection method. The majority chi-square change in the model by order of entry was attributable to TNM stage I-III at diagnosis (Wald chi-square change = 353.10) and least of all to detection method (Wald chi-square change = 8.67) although detection method was significant and retained in the model in last position. The model was adjusted for age, race, and diagnosis year which were not significant Table 2.

**Table 2:** Cox proportional hazards model of distant disease-free interval: outcome = rMBC^a^ (*n* = 6603)

By order of entry into the model:	HzR (95% CI)	*p* value	Wald chi-square	Model Chi-square change	df
TNM Stage I	reference	<.001	200.86	353.10	2
TNM Stage II	3.30 (2.47, 4.42)		64.96		
TNM Stage III	9.19 (6.70, 12.60)		190.03		
HR/HER2 status: initial diagnosis					
HR+/HER2-	reference	<.001	48.50	55.00	2
HR+ or HR−/HER2+	.86 (.66, 1.13)		1.13		
HR−/HER2-	2.22 (1.73, 2.84)		40.31		
Histological grade primary tumor					
Low/Intermediate	reference	<.001	7.89	29.04	1
High	1.49 (1.13, 1.98)				
Detection method					
MamD	reference	<.001	25.84	8.51	1
PtD	1.80 (1.43, 2.25)				

^a^adjusted for age, race and diagnosis year (not significant in the model)

**Fig. 5:**
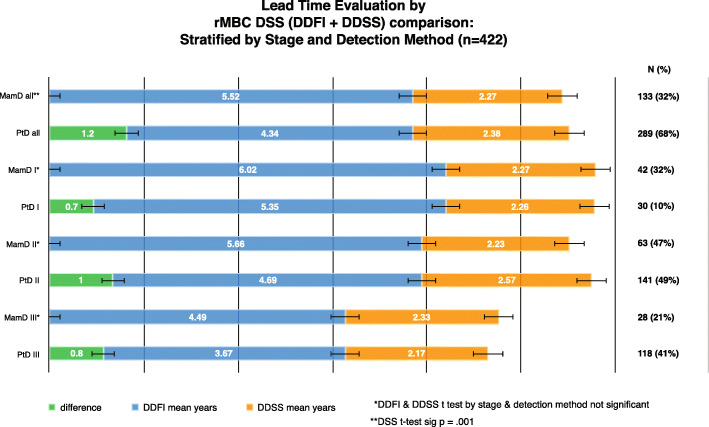
rMBC disease survival time by detection method and DDFI+DDSS stratified by stage and detection method (*n* = 422)

Mean unadjusted time to distant recurrence (DDFI) was significantly different [MamD 5.52 years, PtD 4.34 years (*p* = .001) (difference 1.18 years)] Fig. 5. Unadjusted for detection method mean DDFI interval was significantly different between stages [I = 5.74 years, II = 5.00 years, III = 3.83 years, *p* < .001]. Mean times to distant disease recurrence for PtD and MamD BC stratified by stage to adjust for effect modification differed but were not statistically significantly different [stage I: MamD 6.02 years, PtD 5.35 years (difference 8 months), *p* = .902; stage II: MamD 5.66 years, PtD 4.69 years (difference 12 months), *p* = .537; Stage III: MamD 4.49 years, PtD 3.67 years (difference 10 months, *p* = .597]. Mean time from distant disease recurrence to death or last follow up (DDSS) did not differ by detection method overall [MamD 2.27 years, PtD 2.38 years (*p* = .154)] Fig. 5.

## Discussion

The majority of distant disease recurrence (68%) occurred among patient-detected cases with an incidence rate of 11% compared to 3% distant recurrence among mammography detected breast cancer cases. Mean time to distant recurrence was shorter by 8–12 months for patient-detected BC than it was for mammography-detected BC but did not differ significantly when stratified by the effect modifier stage at diagnosis. Stage at diagnosis was more strongly associated with the outcome distant recurrence than other factors in the model including detection method. There was no difference in time from distant disease recurrence to last follow-up or death from disease by detection method. Breaking down lead time interval by mammography/patient detection and stage revealed that differential lead time was only present in the initial interval from diagnosis to distant recurrence.

Time from initial diagnosis to distant recurrence is the first interval and time from distant recurrence to last follow-up or death is the second interval in disease progression and disease specific survival. Time to disease progression would be equivalent between screening and symptomatic presentation if lead time differences were not present. Although there were differences in DDFI mean time comparisons by stage, the differences were not significant. In the model adjusted for stage, HR/HER2 status and histologic grade, detection method ranked last in the model with a small but significant effect as measured by the Wald chi-square statistic. Lead time interval related to detection method appears to be a less influential factor in distant disease-free interval as other factors related to early diagnosis, stage, HR/HER2 status and histologic grade, represent the majority of effect on distant disease-free interval superseding the effect of detection method.

The survival curves illustrate differential survival by detection method with better mammography detected breast cancer survival. The survival curves of the rMBC patient group illustrate the differential survival which occurs prior to metastatic recurrence with no difference in time from distant disease to death as a function of detection method.

As a result of stage shift over time related to mammography screening, stage at diagnosis, histologic grade, and hormone receptor/HER2 status are not evenly distributed between patient and mammography detected invasive breast cancer cases. Stage at diagnosis is most strongly associated with the outcome, distant disease recurrence, in the Cox proportional hazards model The magnitude of the effect of detection method, on the outcome, length of time to distant recurrence, differs substantially by stage at diagnosis. Stratified analysis adjusts for effect modification and provides an opportunity to measure differential lead time by detection method. The natural ordering of stage at diagnosis lends itself to a linear approach for stratified analysis. From our analysis it appears the difference in survival is associated with the lengthened first interval, time to distant recurrence and not the second interval, time from distant recurrence to death.

### Strengths and limitations

Mammography screening in the United States relies on opportunistic mammography screening based on United States Preventive Services Task Force, the American Cancer Society and other organizations recommendations unlike countries with organized screening programs [23, 24, 31, 32]. Screening is therefore predicated on self-initiation of screening mammography or prompted by a care provider or health care system. In the absence of a national screening program and as screening participation data connected to outcomes is not readily available, we tested the differential lead time hypothesis using an institutional cohort and mammography detected breast cancer as a proxy for screen detection compared to patient detected breast cancer in a real-world setting. Inflammatory breast cancer and physician detected breast cancer, both rare events (< 5%), were not included in the analysis as differential presentation and survival did not contribute to specific hypothesis testing of exposure (mammography detected compared to patient detected BC survival time) and could affect the generalizability of outcomes by diluting the measured effect.

Mammography screening participation rate reported in the year prior to 2012 was 57% in Washington State, very close to our observed rate of 61% mammography detection [33]. We do not have information regarding age appropriate mammography screening program participation or time interval between last non-diagnostic mammogram and breast cancer discovered by mammography. While it has been speculated that some mammography screen detected cancer would not become clinically evident in a woman’s life time, to date there are no published reports of screen detected breast cancer regression or spontaneous disappearance [34]. Only invasive breast cancer stage I-III were included in the analysis as stage 0 may be interpreted as an overdiagnosis category detected by mammography and therefore not compatible with survival comparison by detection method [8].

### Interpretation: comparison to other studies

In a study of 233 patients diagnosed in 1988 patients with mammography screen-detected breast cancer had superior prognosis primarily due to the mammography detected better prognosis characteristics and low stage breast cancer at diagnosis compared to the non-mammography screen detected group [35]. Within stage the mammography detected invasive breast cancers had superior prognosis. The minimal lead time estimate from early screening studies conducted by the Health Insurance Plan of Greater New York was 10 months with estimates from statistical models ranging from 7 to 13 months [17, 18]. Their statistical models suggested the average lead time gained by screening to be about a year. In a more recent study by Allgood et al., it was found the majority of survival advantage of screen detection was due to size and node status [36].

Corrections for and explanation of lead time, or lead time bias as it is called in relation to mammography screening, largely rely on modelling and/or statistical estimates applied to population data and lack information on time to distant recurrence and time to death from distant disease [37–39]. In our institutional cohort with distant recurrence date and distant disease survival time we have a different approach to evaluate lead time using real as opposed to modelled data.

Lead time interval is the time between screening detection and when disease would become clinically evident without screening, assuming the same disease progression post diagnosis regardless of detection method. In the case of invasive breast cancer, it appears lead time advance by mammography detection adds to evident disease progression time extending the time interval to distant recurrence regardless of stage at diagnosis. This may be due to as yet unidentified benefit related to mammography screening [16, 40]. We observed no difference in distant disease survival by detection method indicating lead time only factors in the first time-interval of disease progression and once distant metastatic disease is present disease progression is the same.

### Generalizability

The Seattle-Puget Sound region where the study was conducted has high socioeconomic status (SES) with ready access to care and high insured percentage [41]. Patients treated at this institution may not be comparable to other U. S. geographic areas. Our breast cancer survival rates have been documented by national comparisons to have greater improvement over time than national rates [42].

## Conclusions

From our analysis, mammography detected breast cancer was associated with earlier stage, higher percentage HR positive/HER2 negative subtype and lower histologic grade disease, factors associated with reduced distant recurrence and better outcomes. Earlier stage at diagnosis was the dominant factor affecting better survival, giving mammography detected breast cancer cases an overall survival advantage. However, once distant disease occurred, no distant disease survival time difference was observed in spite of more unfavorable PtD breast cancer initial phenotypes. Time to distant recurrence did not differ significantly by detection method stratified by stage and had marginal significance in distant disease modelling. The combined modelling analysis and comparison of lead times indicates lead time presence but less significance compared to other diagnostic characteristics related to survival.

Lead time interval related to detection method may have been a factor with more significant effect in decades preceding current diagnostic and tumor specific treatment options as over time there has been a shift to earlier stage at diagnosis and declining distant recurrence rates. Without comparative studies of time from diagnosis to distant recurrence and distant recurrence to death from earlier decades, we do not know prior magnitude of effect. Further, lead time interval cannot be measured directly for an entire population, in which the vast majority of patients never suffer a distant recurrence. However, in the modern era of diagnosis and treatment it appears lead time interval related to method of detection while present is not a dominant factor affecting survival relative to other breast cancer characteristics. Early diagnosis, measured by earlier stage breast cancer at diagnosis irrespective of how the breast cancer was detected, is most directly associated with better outcomes and survival. Importantly it appears lead time is a phenomenon related to earlier stage diagnosis by mammography detection. The aim of screening is to find disease at an earlier more treatable stage which screening mammography appears to accomplish.

## Data Availability

The dataset analysed during the current study are not publicly available due HIPAA Security Rules regarding patient data at the institution where the registry was created and is kept and are not available from the corresponding author on reasonable request.

## Abbreviations

BC: Breast cancer
MamD: Mammography detected
PtD: Patient detected
DDFI: Distant disease-free interval
DDSS: Distant disease specific survival
DSS: Disease specific survival
TNM: Tumor, nodes and metastases
HR: Hormone receptor
HER2: Human epidermal growth factor receptor 2
rMBC: Recurrent metastatic breast cancer
IBC: Inflammatory breast cancer
HIPAA: Health insurance portability and accountability act
IRB: Institutional review board
SEER: Surveillance Epidemiology and End Results
AJCC: American Joint Committee on Cancer
HzR: Hazard ratio
CI: Confidence interval
ln: Natural logarithm
TNBC: Triple negative breast cancer
*p*-value: Probability value
